# β,β-Dimethylacrylshikonin sensitizes human colon cancer cells to ionizing radiation through the upregulation of reactive oxygen species

**DOI:** 10.3892/ol.2014.2018

**Published:** 2014-03-31

**Authors:** SEO-YOUNG KWAK, YOUN KYOUNG JEONG, BU-YEON KIM, JI YOUNG LEE, HYUN-JOO AHN, JAE-HOON JEONG, MI-SOOK KIM, JOON KIM, YOUNG-HOON HAN

**Affiliations:** 1 Division of Radiation Cancer Research, Korea Institute of Radiological and Medical Sciences, Nowon-gu, Seoul 139-706, Republic of Korea; 2 Laboratory of Biochemistry, School of Life Sciences and Biotechnology, Korea University, Seoul 136-701, Republic of Korea; 3 Research Center for Radiotherapy, Korea Institute of Radiological and Medical Sciences, Nowon-gu, Seoul 139-706, Republic of Korea

**Keywords:** shikonin, β,β-dimethylacrylshikonin, radiosensitizer, reactive oxygen species, apoptosis

## Abstract

Shikonin, a naphthoquinone derivative, has been shown to possess antitumor activity. In the present study, the effects of shikonin and its analog, β,β-dimethylacrylshikonin, were investigated as radiosensitizers on the human colon cancer cell line, HCT-116. Shikonin and, to a greater extent, its analog-induced apoptosis of HCT-116 cells further synergistically potentiated the induction of apoptosis when combined with ionizing radiation (IR) treatment. Shikonins also stimulated an increase in reactive oxygen species (ROS) production and IR-induced DNA damage. Pre-treatment with the ROS scavenger, *N*-acetylcysteine, suppressed the enhancement of IR-induced DNA damage and apoptosis stimulated by shikonins, indicating that shikonins exert their radiosensitizing effects through ROS upregulation. The radiosensitizing effect of shikonins was also examined *in vivo* using the xenograft mouse model. Consistent with the *in vitro* results, injection of β,β-dimethylacrylshikonin combined with IR treatment significantly suppressed tumor growth of the HCT-116 xenograft. Taken together, the results show that β,β-dimethylacrylshikonin is a promising agent for developing an improved strategy for radiotherapy against tumors.

## Introduction

Shikonin, a naphthoquinone pigment, is the primary component of root extracts from *Lithospermum erythrorhizon*. Shikonin and its analogs have been used for the treatment of burns, measles, sore throat, macular eruption and carbuncles (1). Shikonin and its analogs have also been shown to possess *in vitro* and *in vivo* anticancer activity against various types of cancer (2–4). Shikonins can inhibit tumor growth and prolong the lifespan of tumor-bearing mice (5) and patients with lung cancer (6). Shikonins mediate apoptosis through multiple mechanisms, including induction of the generation of reactive oxygen species (ROS) (7,8) and cell cycle arrest via a caspase-dependent mechanism (9). In addition, shikonin exhibits antiangiogenic activity (10) and can also regulate the activity of topoisomerase I and II, leading to DNA cleavage (11,12).

Along with surgery and chemotherapy, radiotherapy is one of the most significant modalities for cancer treatment. The use of radiotherapy is primarily limited by intrinsic or acquired resistance to ionizing radiation (IR). In an effort to overcome the radioresistance of cancer cells to improve radiotherapy, a variety of chemical compounds have been tested for their radiosensitizing effects. Curcumin (13–15), resveratrol (16), genistein (17–19) and flavopiridol (20) have been shown to exhibit radiosensitizing effects on a variety of cancer cells. IR kills cancer cells by inducing DNA damage and generating ROS, which in turn induces further damage of biomolecules, including DNA. The accumulation of ROS also induces the deregulation of the apoptotic signaling pathway, ultimately leading to apoptosis. The radiosensitizing effect of compounds is often associated with ROS upregulation, indicating that the ROS-mediated mechanism may be a significant target for achieving biological enhancement of the effects of radiotherapy (21).

Although shikonin and its derivatives have been reported to have potential anticancer activity, they have not been examined for their effects on radiotherapy. The present study examined whether shikonin and its analog, β,β-dimethylacrylshikonin, exhibit radiosensitizing effects, and investigated the possible utilization of these compounds as radiotherapy-enhancing agents.

## Materials and methods

### Cell culture

The HCT-116, H460 and A549 cells obtained from the American Type Culture Collection (Manassas, VA, USA) were grown in RPMI-1640 medium (Mediatech, Manassas, VA, USA) and LN428 was grown in MEM (Mediatech) supplemented with 10% fetal bovine serum (Tissue Culture Biologicals, Los Alamitos, CA, USA), penicillin/streptomycin (1X; PAA Laboratories GmbH, Morningside, QLD, Australia) and mycokill (5 mg/ml, PAA laboratories). Cells were maintained at 37°C in a humidified incubator containing 5% CO_2_. Subconfluent cells were treated with shikonin (Biomol Research Laboratories, Inc., Plymouth Meeting, PA, USA) or its analogue, β,β-dimethylacrylshikonin (Tokyo Chemical Industry Co., Ltd., Tokyo, Japan) for 4 h followed by IR treatment at 5 Gy for the indicated time.

### Growth inhibition assay

Cells were seeded in 96-well plates and pre-treated with shikonins for 4 h, and subsequently exposed to IR at the indicated doses. The number of viable cells was determined using the 3-(4,5-dimethylthiazol-2-yl)-5-(3-carboxymethoxyphenyl)-2-(4-sulfophenyl)-2*H*-tetrazolium, inner salt (MTS) reduction assay (CellTiter 96 AQueous Non-Radioactive Cell Proliferation assay; Promega, Madison, WI, USA) according to the manufacturer’s instructions. The experiments were carried out in triplicate.

### Colony formation assay

Cells were seeded in 60-mm dishes at a density of 500 cells per dish. Shikonins were added to each dish 4 h prior to IR treatment. After 14 days, media were removed, then cells were stained with 1% crystal violet (Sigma-Aldrich, St. Louis, MO, USA) in 10% ethanol and counted. The experiments were carried out in triplicate.

### Apoptosis assay

Cells were pre-treated with shikonins (0.5 μM) for 4 h and irradiated. The media were then exchanged with fresh media and the cells were incubated for 72 h, followed by Annexin V/propidium iodide (PI)-double staining using Annexin V-FITC Apoptosis Detection kit I (BD Biosciences, Franklin Lakes, NJ, USA). For the ROS scavenging experiment, *N*-acetylcysteine (NAC; Sigma-Aldrich) was pre-treated at 1 mM for 2 h prior to the treatment with shikonins. Cell death was analyzed using a fluorescence-activated cell sorting (FACS)Calibur apparatus (BD Biosciences).

### Determination of intracellular ROS level

Intracellular ROS production was measured by staining cells with the fluorescent probe, 2′-7′-dichlorofluoresceindiacetate (DCF-DA; Invitrogen Life Technologies, Carlsbad, CA, USA). The cells that were treated with a combination of shikonins and IR were incubated with DCF-DA at 1 μM for 30 min. The changes in fluorescence intensity were monitored by flow cytometry using a FACSCalibur apparatus (BD Biosciences).

### Western blot analysis

Following drug treatment, cell lysates were prepared for western blot analysis. Proteins were resolved by SDS-polyacrylamide gel electrophoresis (Bio-Rad, Hercules, CA, USA) and transferred to nitrocellulose membrane (Whatman, Pittsburgh, PA, USA). Subsequent to the transfer, the membranes were blocked in 5% skimmed milk in 10 mM Tris-HCl (pH 8.0), 150 mM NaCl and 0.05% Tween-20 (TBST; Amresco, Solon, OH, USA) for 30 min, and then incubated with a primary antibody in 5% skimmed milk in TBST for 2 h at room temperature. The membranes were washed three times with TBST and incubated for 1 h in TBST containing horseradish peroxidase-linked anti-immunoglobulin G (IgG). Following three washes in TBST, immunoreactive products were detected by chemiluminescence (ECL Plus; Amersham Pharmacia Biotech, Piscataway, NJ, USA). Mouse monoclonal anti-γH2AX and anti-β-actin antibodies were purchased from Millipore (Billerica, MA, USA) and Santa Cruz Biotechnology, Inc. (Santa Cruz, CA, USA), respectively.

### Ionizing irradiation of cells

Cells were exposed to γ-rays with a ^137^Cs γ-ray source (Atomic Energy of Canada, Ltd., Ontario, ON, Canada) and a dose rate of 2.6 Gy/min.

### Immunofluorescence microscopy

The cells were seeded on a cover glass in 24-well plates. The media were removed and carefully rinsed with phosphate-buffered saline (PBS) 30 min following the treatments with shikonins and IR. The cells were fixed with 3.7% paraformaldehyde in PBS for 10 min and washed twice with PBS. Cells were permeabilized for 10 min with 0.1% Triton X-100 followed by blocking with CAS-block (Invitrogen Life Technologies) for 30 min. Cells were then stained by incubating with mouse monoclonal anti-γH2AX antibody (1:500 dilution) followed by goat anti-mouse IgG-Alexa Fluor555 (Invitrogen Life Technologies) (1:1000 dilution). 4,6-Diamidino-2-phenylindole (DAPI) (50 μg/ml) was added to the secondary antibody mixture to visualize the nuclei. Fluorescence images were obtained using a LSM710 confocal microscope (Carl Zeiss Group, Jena, Germany).

### Tumor xenograft growth in athymic mice

Athymic nude mice (4-week-old males) were obtained from Orientbio, Inc., (Seoul, South Korea) and were maintained in a laminar air-flow cabinet under specific pathogen-free conditions. The human colon cancer HCT-116 xenograft mouse model was established by subcutaneous inoculation of 2×10^6^ cells into the right hind leg. When the tumor size reached 150 mm^3^, the mice were randomly divided into six groups (seven mice per group) and treated with either the vehicle (10% dimethylsulfoxide in PBS) or shikonins (2.0 mg/kg) in the presence or absence of IR. Two days after treatment, the second injection was prepared. Locoregional irradiation was applied in single 8-Gy doses using a Co-60 irradiator (Theratron 780; Atomic Energy of Canada). Two perpendicular diameters of tumors were measured twice a week with a caliper square by the same investigator, and the tumor volume was calculated using the following equation: Tumor volume (V) mm^3^ = (smaller diameter)^2^ × (larger diameter) × (π/6). The experiment was terminated when the tumor volume in the control group reached 3000 mm^3^. All animal protocols were reviewed using the Good Laboratory Practice guidelines of the Radiotherapy Research Center, Korea Institute of Radiological and Medical Sciences (Seoul, Korea). The use of these animals and the experimental procedures were approved by the Institutional Animal Care and Use Committee of the Korea Institute of Radiological and Medical Sciences.

### Statistical analysis

All data were plotted in terms of mean ± standard error of the mean values. Statistical analysis was assessed using a parametric repeated-measures one-way analysis of variance followed by Tukey’s multiple comparison test (Graph Pad version 3; San Diego, CA, USA). A value of P<0.05 was considered to indicate a statistically significant difference.

## Results

### Shikonins sensitize cancer cell lines to IR

**Figure 1:**
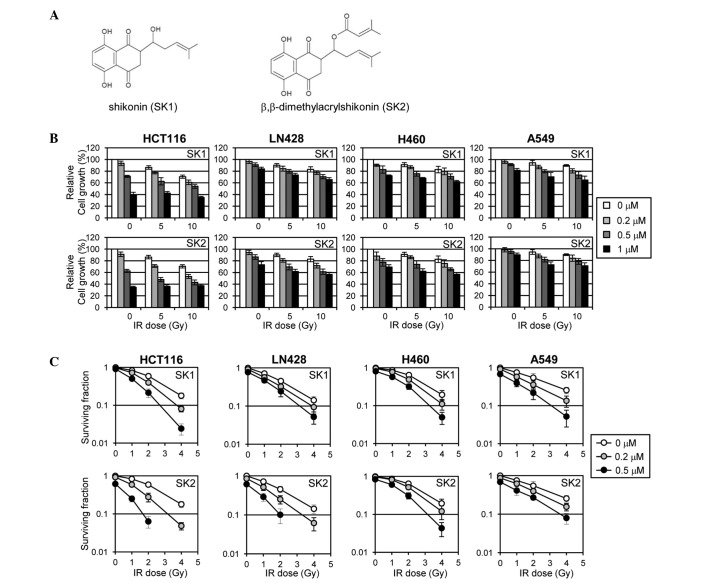
Shikonins sensitize cancer cell lines to IR treatment. (A) Chemical structures of shikonin and β,β-dimethylacrylshikonin. (B) MTS assay: The cells were pre-treated with shikonins (0, 0.2, 0.5 and 1 μM) for 4 h and exposed to IR (0, 5 and 10 Gy). The viability of cells was assessed using the MTS assay kit after 72 h. (C) Colony-formation assay: The cells were treated with shikonins and IR as aforementioned. After 14 days, the colonies were stained with 1% crystal violet in 10% ethanol and counted. The experiments were performed in triplicate. IR, ionizing radiation; MTS, 3-(4,5-dimethylthiazol-2-yl)-5-(3-carboxymethoxyphenyl)-2-(4-sulfophenyl)-2H-tetrazolium, inner salt.

To investigate the effect of shikonin analogs on the cancer cell response to IR, shikonin and its analog β,β-dimethylacrylshikonin were selected for the present study and their chemical structures are shown in Fig. 1A. First, the effect of shikonins on the proliferation of cancer cells when used alone or in combination with IR was determined. For this experiment, various cancer cell lines, including HCT-116 (colon cancer), LN428 (glioma), H460 (lung cancer) and A549 (lung cancer) cells, were used. The cells were pre-treated with shikonins for 4 h and irradiated at the indicated doses in Fig. 1B. The viability of cells was determined using the MTS assay. Shikonins inhibited the overall proliferation of the cell lines in a dose-dependent manner and exhibited additional effects when combined with IR (Fig. 1B). Among the cell lines examined, HCT-116 was the most sensitive to shikonin treatment with respect to inhibition of proliferation, as determined by the MTS assay. Subsequently, the effect of shikonins on the cellular response to IR was evaluated by determining clonogenic cell survival following IR treatment through a colony formation assay. Shikonin exhibited a moderate radiosensitizing effect for HCT-116, but minor effects for the other cell lines, while the radiosensitizing effect of β,β-dimethylacrylshikonin was considerable for HCT-116 and LN428, but minor for H460 and A549 (Fig. 1C). Overall, these data show that shikonins sensitize HCT-116 cells more efficiently to IR treatment and, therefore, the HCT-116 cells were used for further in-depth study of the radiosensitizing effects of shikonins.

### Shikonins enhance IR-induced apoptosis

**Figure 2:**
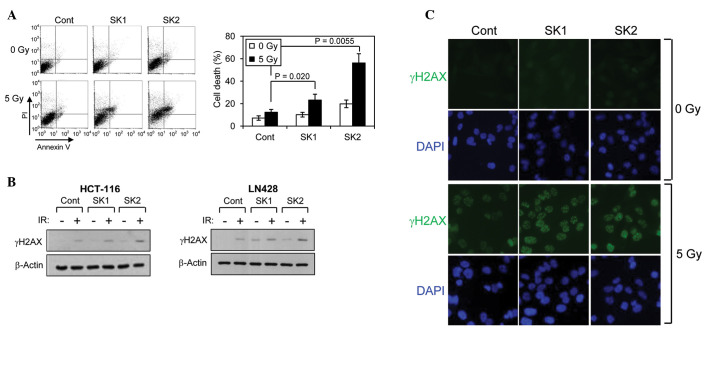
Shikonins enhance IR-induced apoptosis and DNA damage in HCT-116 cells. (A) The cells were pre-treated with shikonins (0.5 μM) for 4 h and exposed to IR at 5 Gy. After 48 h, the apoptotic cells were determined by Annexin V/PI staining. (B) Determination of DNA damage by western blot analysis for the phosphorylated H2AX (γH2AX). (C) Visualization of γH2AX foci formation. Shikonin-treated cells were exposed to IR, fixed after 30 min and processed for immunofluorescence staining. IR, ionizing radiation; PI, propidium iodide; DAP1, 4,6-diamidino-2-phenylindole; SK1, shikonin; SK2, β,β-dimethylacrylshikonin.

The induction of apoptosis in HCT-116 cells was analyzed following combined treatment of shikonins with IR. The cells were irradiated following pre-treatment or no pre-treatment with shikonins and were analyzed for apoptosis by Annexin V/PI staining at 72 h following irradiation. The cells that stained negative for Annexin V and PI were assigned as undamaged live cells. Shikonin induced marginal cell death, and the extent of further enhancement of cell death by combination with IR was not significant (Fig. 2A). By contrast, treatment with β,β-dimethylacrylshikonin alone induced significant cell death, and further enhancement of cell death was observed when combined with IR. These results indicate that β,β-dimethylacrylshikonin is extremely effective and more effective compared with shikonin in rendering HCT-116 cells more susceptible to IR-induced cell death. It has great potential as a radiosensitizing agent.

### Shikonins enhance IR-induced DNA damage

The effect of shikonins on the extent of IR-induced DNA damage was examined by determining the level of the phosphorylated histone H2AX (γH2AX), a well-known marker for DNA double-strand breaks. Single treatment with either shikonin or β,β-dimethylacrylshikonin caused weak accumulation of γH2AX in HCT-116 and LN428 cells, indicating that shikonin and β,β-dimethylacrylshikonin can individually induce DNA damage to a certain extent. However, when the cells were treated with a combination of shikonins and IR, only β,β-dimethylacrylshikonin strongly enhanced further IR-induced γH2AX increases (Fig. 2B). The effect of shikonins on the induction of DNA damage was also assessed by visualizing γH2AX foci with immunofluorescence microscopy. Treatment with either of the shikonins increased the formation of γH2AX foci, but β,β-dimethylacrylshikonin-treated cells showed a stronger γH2AX signal intensity compared with shikonin-treated cells following exposure to IR (Fig. 2C). These results indicate that β,β-dimethylacrylshikonin strongly potentiates the induction of DNA damage by IR treatment and that this potentiation is greater compared with that observed with shikonin.

### Combined treatment of shikonins and IR causes ROS accumulation

**Figure 3:**
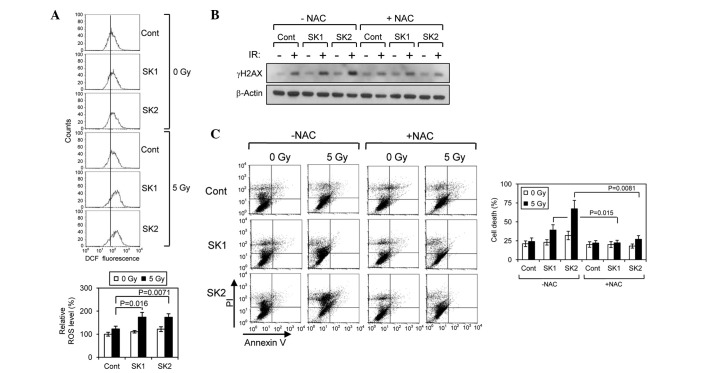
Shikonins enhance IR-induced apoptosis through ROS upregulation. (A) Determination of the cellular ROS level by staining with DCF-DA followed by FACS analysis. The cells were treated with shikonins (0.5 μM) in the presence or absence of IR treatment (5 Gy), and the ROS level was measured by FACS analysis. (B) Suppression of the effect of shikonins on H2AX phosphorylation by NAC pretreatment. (C) Suppression of the effect of shikonins on IR-induced apoptosis by NAC pre-treatment. IR, ionizing radiation; NAC, *N*-acetylcysteine; ROS, reactive oxygen species; PI, propidium iodide; DCF-DA, 2′-7′-dichlorofluoresceindiacetate; FACS, fluorescence-activated cell sorting; SK1, shikonin; SK2, β,β-dimethylacrylshikonin.

ROS generation is one of the primary mechanisms by which IR kills cells, and it has been reported that shikonin causes apoptosis through an ROS/c-Jun N-terminal kinase-mediated signaling pathway in the breakpoint cluster region/Abelson-positive chronic myelogenous leukemia cells (8). Therefore, it was initially postulated that shikonins modulate the cellular response to IR through the regulation of ROS levels. To investigate this possibility, the effect of a combined treatment of shikonins and IR at the intracellular ROS level was examined. A single IR treatment (5 Gy) with either of the shikonins caused a minor increase (~15–25%) in ROS levels in the HCT-116 cells. However, IR treatment following pre-treatment with either of the shikonins resulted in a significant increase (~80%) in ROS levels (Fig. 3A). This synergistic effect of combined treatment of shikonins and IR on ROS accumulation indicates that shikonins may predispose cancer cells to accumulate more ROS in response to IR treatment.

### ROS is involved in the synergistic effect of shikonins on IR-induced DNA damage and cell death

The preceding observations that shikonins enhance ROS accumulation, DNA damage and apoptosis indicate that ROS accumulation may be responsible for the synergistic effect of shikonins on DNA damage and subsequent cell death. To test this hypothesis, the effect of pretreatment of cells with NAC on ROS-induced DNA damage was examined, which can be assessed by determining the level of γH2AX (Fig. 3B). NAC significantly attenuated the increase in the γH2AX level induced by either of the shikonins. NAC also significantly suppressed the synergistic effect of β,β-dimethylacrylshikonin on IR-induced apoptosis (Fig. 3C). These observations indicate that ROS accumulation plays a critical role in the enhancement of IR-induced DNA damage and subsequent apoptosis by β,β-dimethylacrylshikonin treatment.

### β,β-Dimethylacrylshikonin potentiates the antitumor effect of IR on tumor growth in the HCT-116 xenograft mouse model

**Figure 4:**
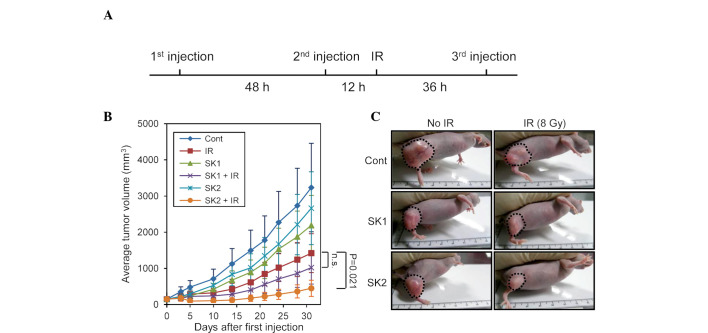
Combination of β,β-dimethylacrylshikonin with IR significantly suppresses tumor growth in HCT-116 xenograft mice. (A) Scheme for the *in vivo* experimental procedure. (B) Change in calculated tumor volume at the indicated intervals. A value of P<0.05 was considered statistically significant. (n=6). (C) Photographs showing the tumor size endpoint. IR, ionizing radiation; SK1, shikonin; SK2, β,β-dimethylacrylshikonin.

To validate the radiosensitizing effect of shikonins *in vivo*, the HCT-116 xenografts in athymic nude mice were established. Using the experimental procedure described in Fig. 4A, the change in tumor volume was monitored twice a week following the combined treatment with the shikonins and IR. While tumor growth was moderately suppressed by IR alone, β,β-dimethylacrylshikonin completely retarded tumor growth when coupled with IR treatment (Fig. 4B). The tumor size endpoint, which was measured 30 days subsequent to IR treatment when the tumor volume of the vehicle group reached 3000 mm^3^, also manifested the strong effect of β,β-dimethylacrylshikonin acting synergistically with IR to suppress tumor growth (Fig. 4C).

## Discussion

Radiotherapy is one of the primary modalities in cancer treatment and is generally used in combination with surgery or chemotherapy (22). The use of high-dose IR also inevitably causes damage to surrounding normal tissues, necessitating the use of agents to sensitize cancer cells to IR treatment, thereby allowing the use of lower doses of radiation. In spite of numerous reports that have demonstrated the antitumor effect of shikonins (2–4), the potential applicability of shikonins as radiosensitizers has not been fully examined. In an effort to identify novel radiosensitizers, the effect of shikonin and its analog β,β-dimethylacrylshikonin on the sensitivity of cancer cells to IR treatment was examined.

IR-induced cell death was promoted by pre-treatment with shikonin or more strongly with β,β-dimethylacrylshikonin. Synergistic increases in intracellular ROS levels and DNA damage accompanied the IR-sensitizing action of shikonins. It was also found that the enhancement of IR-induced DNA damage and cell death mediated by shikonins was abolished in the presence of the antioxidant NAC. Since the generation of ROS is one of the primary mechanisms by which IR induces DNA damage and kills cells, these results indicate that further upregulation of ROS to intolerable levels accounts for the radiosensitizing effects of shikonins. A recent study using leukemia cells indicated that the cytotoxicity of shikonin involves the disruption of mitochondrial function, including ROS production and the inhibition of cytoskeleton formation (23). Shikonin immediately accumulates in the mitochondria and disrupts the mitochondrial membrane potential, followed by the induction of oxidative damage due to the generation of ROS. Several studies have demonstrated the antitumor activity of β,β-dimethylacrylshikonin via various signaling pathways, including the extracellular signal-regulated kinase and Notch-1 pathways (24–26). Notably, β,β-dimethylacrylshikonin has been reported to inhibit the cellular growth of HCT-116 cells *in vitro* and of xenografts *in vivo* (26). A previous study showed that the induction of apoptosis by β,β-dimethylacrylshikonin is associated with the upregulation of the proapoptotic proteins, Bax and Bid, and a reduction in the expression of the antiapoptotic proteins, B-cell lymphoma 2 (Bcl-2) and Bcl-XL (27). This change in the ratio of the proapoptotic/antiapoptotic Bcl-2 family of proteins may have led to ROS generation. These observations are in accordance with the overall results in the present study that demonstrated the ROS-mediated radiosensitizing effect of shikonins.

In summary, the present study has demonstrated significant radiosensitizing activity of β,β-dimethylacrylshikonin *in vitro* and *in vivo*. These findings indicate that β,β-dimethylacrylshikonin is a promising candidate for a radiosensitizing agent and may be exploited for the development of a novel strategy for improving radiotherapy against cancerous tumors.
